# Application of Plasmonic Metal Nanoparticles in TiO_2_-SiO_2_ Composite as an Efficient Solar-Activated Photocatalyst: A Review Paper

**DOI:** 10.3389/fchem.2020.568063

**Published:** 2021-01-29

**Authors:** Collin G. Joseph, Yun Hin Taufiq-Yap, Baba Musta, Mohd Sani Sarjadi, L. Elilarasi

**Affiliations:** ^1^ Sonophotochemistry Research Group, Faculty of Science and Natural Resources, Universiti Malaysia Sabah, Kota Kinabalu, Sabah; ^2^ Water Research Unit, Faculty of Science and Natural Resources, Universiti Malaysia Sabah, Kota Kinabalu, Sabah; ^3^ Industrial Chemistry Programme, Faculty of Science and Natural Resources, Universiti Malaysia Sabah, Kota Kinabalu, Sabah; ^4^ Chancellery Office, Universiti Malaysia Sabah, Kota Kinabalu, Sabah; ^5^ Catalysis Science and Technology Research Centre, Faculty of Science, Universiti Putra Malaysia, Serdang, Malaysia; ^6^ Centre of Foundation, Language and Malaysian Studies, International University of Malaya-Wales, Kuala Lumpur, Malaysia

**Keywords:** TiO_2_-SiO_2_, plasmonic metal nanoparticles, visible region, photocatalysis, dye

## Abstract

Over the last decade, interest in the utilization of solar energy for photocatalysis treatment processes has taken centre-stage. Researchers had focused on doping TiO_2_ with SiO_2_ to obtain an efficient degradation rate of various types of target pollutants both under UV and visible-light irradiation. In order to further improve this degradation effect, some researchers resorted to incorporate plasmonic metal nanoparticles such as silver and gold into the combined TiO_2_-SiO_2_ to fully optimize the TiO_2_-SiO_2_’s potential in the visible-light region. This article focuses on the challenges in utilizing TiO_2_ in the visible-light region, the contribution of SiO_2_ in enhancing photocatalytic activities of the TiO_2_-SiO_2_ photocatalyst, and the ability of plasmonic metal nanoparticles (Ag and Au) to edge the TiO_2_-SiO_2_ photocatalyst toward an efficient solar photocatalyst.

## Introduction

The first breakthrough in producing the photocatalytic effect of TiO_2_ was reported by Fujishima-Honda in 1972, in which, photosplitting of water in the presence of TiO_2_ was achieved. The team documented the generation of oxygen gas bubbles at the electrode containing TiO_2_ placed under an electrical contact with a piece of platinum metal. While both were immersed in water and exposed to light, hydrogen gas was detected at the platinum electrode. The explanation for this effect was that TiO_2_, under ultraviolet irradiation with a wavelength lower than 380 nm, produces an electron-hole pairs according to the following equation:

$${\text{TiO}}_{2}+\mathrm{hv}\to {\text{TiO}}_{2}\left(e_{CB^{-}}+h_{VB^{+}}\right)$$ (1)

The electron-hole pairs diffuse evenly on the surface of the TiO_2_ particle, to react and decompose oxygen and water present in the atmosphere in order to produce HO^•^, hydroxyl radicals, and O^−^_2_, superoxide ions, according to the following equations:

$${\text{TiO}}_{2}\left({\text{h}}_{{\text{VB}}^{+}}\right){+\text{H}}_{2}\text{O}\to {\text{TiO}}_{2}{+\text{H}}^{+}+{\text{OH}}^{-}$$ (2)

$${\text{TiO}}_{2}\left(h_{VB^{+}}\right)+{\text{OH}}^{-}\to {\text{TiO}}_{2}+\text{OH}$$ (3)

$${\text{TiO}}_{2}\left(e_{CB^{-}}\right)+{\text{O}}_{2}\to {\text{TiO}}_{2}+{\text{O}}_{2}^{-}$$ (4)

These oxidants will disintegrate and restructure the pollutants through redox reactions taking place on the surface of catalyst, into H_2_O, CO_2_, and mineral acids (Zangeneh et al., 2015). The mineral acids are generated due to presence of heteroatoms, i.e., S, N, and Cl in the organic compounds (Gardin et al., 2010). TiO_2_, is a commonly used photocatalyst due to its availability, its efficient photoactivity, highest chemical stability, harmless nature, lowest cost, and ability in mineralizing various organic contaminants including dyes, insecticides, aromatics, alkanes, haloalkanes, alcohols, and surfactants (Gardin et al., 2010; Abdullah et al., 2016).

**FIGURE 1:**
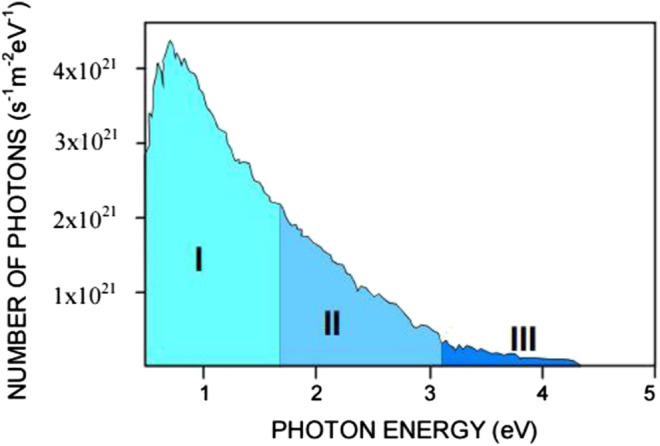
The solar energy spectrum based on the number of photons received per second per unit area of 1 m^2^ vs. the photon energy detected on a clear sunny day with the sun at 60° above the horizon. I: IR region, II: visible region, and III: UV region cited by Choi, 2007 which was originally adapted from Oriel-Instruments (Book of Photon Tools, Oriel-Instruments, 1999).

TiO_2_-based photocatalysis has long been utilized as one of the methods to remediate wastewater. The main obstruction for the application of TiO_2_ under solar energy is its high recombination rate of photoexcited electron-hole pairs and wide band gap which is 3.2 eV (Liu et al., 2013). Due to its wide band gap, it requires photon energy equals or higher than 3.2 eV, in order to induce the photoexcitation of electrons and holes. Unfortunately, majority of photons in visible region have photon energy less than 3.0 eV (based on Figure 1). In order to overcome these restrictions, TiO_2_ is prepared as a composite catalyst in which the newly added material enables the utilization of TiO_2_ in the visible-light region. For instance, doping CuBiS_2_, a p-type semiconductor (with a band-gap value of 2.19–2.62 eV) onto TiO_2_, enabled the direct utilization of TiO_2_ photocatalytic activity in the visible-light region (Abdullah & Kuo, 2015).

Another hindrance in utilizing TiO_2_ photocatalysis on an industrial scale is the requirement of a large amount of energy to power the UV lamps, resulting in its high operating costs (Chan et al., 2012). In order to overcome these drawbacks, employing sunlight energy to power the photoexcitation of the electrons and holes in the TiO_2_ semiconductor would be most ideal. The tapping of solar energy to drive the remediation of wastewater by photocatalysis processes will enable the classification of conventional photocatalysis methods as renewable energy with green technology characteristics.

Due to these benefits, many investigators are currently working on extending the application of TiO_2_ photocatalysis into visible-light region. In order to achieve this, one of the methods focused on is the synthesizing of SiO_2_-modified TiO_2_ photocatalysts which have shown remarkable efficiency under visible-light region.

## TiO_2_-SiO_2_ Photocatalysis

### Effect of SiO_2_ on Specific Surface Area of TiO_2_-SiO_2_ Composite

SiO_2_ is typically used as a catalyst support or dopant dispersed within the TiO_2_ lattice (Liga et al., 2013). This doping affects the TiO_2_’s fundamental properties and thus influences the photocatalytic activities too. The structure modification of TiO_2_ by SiO_2_ drastically increases the specific surface area of the TiO_2_-based photocatalyst. The high surface area of TiO_2_-SiO_2_ is due to its high porosity. Structures with high porosity have large internal surface area per weight, and it is this property which provides high accessibility and diffusivity in order to allow molecules to penetrate through pores, resulting in higher degradation of pollutants on the catalyst’s surface (Jeon et al., 2015).

The morphological improvement by SiO_2_ on the TiO_2_’s surface is evident from the literature. Balachandran et al. (2014) reported that the BET specific surface area increased from 65 m^2^ g^−1^ for TiO_2_ to 75 m^2^ g^−1^ for TiO_2_-SiO_2_ while the mean pore size calculated from BET isotherms was 10 nm for TiO_2_ and 15 nm for TiO_2_-SiO_2_. Bellardita et al. (2010) reported specific surface area of TiO_2_-Cabot SiO_2_, TiO_2_-Axim SiO_2_, and TiO_2_-Fly Ash SiO_2_ as 177, 49, and 29 m^2^ g^−1^, respectively. Fatimah et al. (2015) reported TiO_2_-SiO_2_ produced by using rice husk ash as the precursor for SiO_2_ with a specific surface area of 91.91 m^2^/g with a pore volume of 15.18 cc/g compared to that of rice husk ash with a specific surface area of 25.09 m^2^g^−1^ and pore volume of 11.60 cc g^−1^. These results were reportedly higher than that of TiO_2_-SiO_2_ synthesized by using tetraethyl orthosilicate (TEOS) as the SiO_2_ precursor, resulting in catalyst material with a specific surface area of 59.22 m^2^/g and pore volume of 14.92 cc/g. Table 1 shows the BET surface area and crystallite size values of TiO_2_-SiO_2_ photocatalyst reported by Ramamoorthy et al., 2016.

**TABLE 1:** BET surface area and crystallite size values of TiO_2_-SiO_2_ catalysts.

Photocatalyst	BET surface area (m^2^ g^−1^)	Crystallite size (nm)
TiO_2_	92	18
SiO_2_	289	NA
10% TiO_2_-SiO_2_	282	9
20% TiO_2_-SiO_2_	279	10
30% TiO_2_-SiO_2_	279	10
40% TiO_2_-SiO_2_	272	11
50% TiO_2_-SiO_2_	264	12
1% Ag/30% TiO_2_-SiO_2_	274	10

Data in Table 1 clearly indicate that the high surface area of TiO_2_-SiO_2_ is attributed to the high surface area of SiO_2_ itself. SiO_2_ can be synthesized easily via sol gel with a large surface area and pore volume (Ren et al., 2010; Bahadur et al., 2012) and later can be added into TiO_2_ sol, generating composite photocatalyst with increased surface area and pore volume. The enhanced surface area of TiO_2_-SiO_2_ definitely facilitates in achieving higher photocatalytic activity under solar irradiation.

### Effect of SiO_2_ on Crystalline Size of TiO_2_-SiO_2_ Composite

Deposition of SiO_2_ onto TiO_2_ reduces the overall particle size of TiO_2_-SiO_2_ composite particles. Based on the spectra from the UV-Vis spectrophotometer, Balachandran et al. (2014) reported absorption peak of maximum absorbance of TiO_2_ and TiO_2_-SiO_2_ at 372 and 352 nm, respectively. The blue shift indicates decrease in particle size due to quantum confinement effect. As shown in Table 1, Ramamoorthy et al. (2016) reported the reduction in crystallite size of TiO_2_-SiO_2_ (9–12 nm) compared to TiO_2_ (18 nm). In addition, using data from the SEM and TEM analysis, Balachandran et al. (2014) reported that pure TiO_2_ showed irregular morphological structure due to the agglomeration of its particles and has an average diameter of 15–20 nm. Meanwhile, TiO_2_-SiO_2_ showed regular morphology with an average particle size of 7–10 nm. This proves that the SiO_2_-modified TiO_2_ photocatalyst consists of smaller particles but with larger surface area.

The reduced size of TiO_2_-SiO_2_ particles reduces the pathway in which the photoinduced electrons and holes are used to migrate to the active sites on the TiO_2_ surface. This increases the efficiency of the redox reactions by electrons and holes while reducing the recombination rate of photoinduced electrons and holes, thus making TiO_2_-SiO_2_ a better photocatalyst as compared to TiO_2_.

### Effect of SiO_2_ on Surface Acidity of TiO_2_-SiO_2_ Composite

Many previous researchers have reported high surface acidity of TiO_2_-SiO_2_ composite which aids in enhancing the photo decomposition of pollutant molecules. According to Wei et al. (2014), TiO_2_ exhibits Lewis acidity and SiO_2_ does not exhibit any acidity while TiO_2_-SiO_2_ exhibits both Bronsted and Lewis acidity. For TiO_2_-SiO_2_ with TiO_2_ as the main component, the Lewis acid sites are dominant while the Bronsted acid sites are dominant for TiO_2_-SiO_2_ with SiO_2_ as the major component (Fateh et al., 2013). It should be noted that the Ti/Si atomic ratio can be manipulated to control the acidity of the TiO_2_-SiO_2_. TiO_2_-SiO_2_ with atomic ratio of four (Ti:Si = 4) exhibits highest Lewis acidity while TiO_2_-SiO_2_ with atomic ratio of one (Ti:Si = 1) exhibited highest Bronsted acidity (Wei et al., 2014).

The Ti-O-Si bond, a strong acidic bond, in the TiO_2_-SiO_2_ composite photocatalyst, results in a charge imbalance due to the different coordination numbers of the Ti and Si metal center. To offset the negative imbalanced charges over Ti-O, a great deal of protons is extracted from H_2_O molecules generating HO^−^ groups, which in turn enhances the surface acidity. The decomposition rate of pollutant molecules is enhanced due to higher amount of hydroxyl groups being on the surface of TiO_2_-SiO_2_, representing a better photocatalytic performance in visible region compared to TiO_2_ (Kibombo et al., 2012; Xu et al., 2015).

The increased surface acidity attracts and adsorbs more hydroxyl groups which later act as hole-scavengers and readily oxidize the adsorbed H_2_O molecules. These surface hole-scavenger active sites effectively increase the charge separation and reduce the recombination of photoinduced electrons and holes (Kibombo et al., 2012).

### Effect of SiO_2_ on Band Gap of TiO_2_-SiO_2_ Composite

One of the main drawbacks of TiO_2_ is the fast recombination of photoinduced electrons and holes which decreases the efficiency of its photocatalytic activity. Deposition of SiO_2_ onto TiO_2_ caused an increase in the band-gap value of the TiO_2_-SiO_2_ composite, which is due to the quantum-size effect resulting in an increase in the band-gap value and the interface interaction between the oxide phases, either an SiO_2_ matrix or SiO_2_ support effect. The interface interaction leads to a formation of Ti-O-Si bonds strongly modifying the electronic structure of the Ti atoms (Panayotov and Yates, 2003).

As an example, the band-gap value of TiO_2_ and TiO_2_-SiO_2_ as reported by Balachandran et al. (2014) was 3.3 and 3.54 eV, respectively. Bellardita et al. (2010) documented the band-gap value of pure TiO_2_, TiO_2_-Cabot SiO_2_, and TiO_2_/Axim SiO_2_ as 3.00, 3.02, and 2.98 eV, respectively. The increased band gap in TiO_2_-SiO_2_ indicates that the electrons and holes possess stronger reduction (Kibombo et al., 2012) and oxidation abilities and these abilities enhance the photocatalytic activity of the TiO_2_-SiO_2_ photocatalyst in visible region.

### Effect of SiO_2_ on Thermal Stability of TiO_2_-SiO_2_ Composite

Addition of SiO_2_ onto TiO_2_ increases the overall thermal stability of TiO_2_-SiO_2_, thus preventing the conversion from anatase TiO_2_ into rutile TiO_2_ crystalline structure (Kibombo et al., 2012). Table 2 shows the specific surface and pore volume of CTS-1 (Ti:Si = 1), CTS-4 (Ti:Si = 4), and TiO_2_ as a function of the calcination temperature reported by Wei et al. (2014).

**TABLE 2:** Specific surface area and pore volume of CTS-1, CTS-4, and TiO_2_ in relation to calcination temperature (source: Wei et al. (2014)).

Calcination temperature (°C)	CTS-1		CTS-4		TiO_2_	
	Specific surface area (m^2^/g)	Pore volume (ml/g)	Specific surface area (m^2^/g)	Pore volume (ml/g)	Specific surface area (m^2^/g)	Pore volume (ml/g)
500	437	1.39	222	0.50	94	0.33
650	334	0.88	246	0.57	BDL	0.03
800	287	0.75	205	0.57	BDL	BDL^a^
950	176	0.52	121	0.30	BDL	BDL

^a^ BDL = below detection limit.

When the calcination temperature increased from 500 to 950°C, the specific surface area decreased from 437.4 to 176.3 m^2^ g^−1^ for CTS-1 and from 309.5 to 121.2 m^2^ g^−1^ for CTS-4. When the calcination temperature was increased from 500 to 950°C, the pore volume of CTS-1 changed from 1.39 to 0.52 ml g^−1^ and the pore volume of CTS-4 changed from 0.55 to 0.30 ml g^−1^. Meanwhile, for TiO_2_, both specific surface area and pore volume were undetectable after calcination at 650°C or higher due to sintering of particles and collapse of interstitial pores. However, both CTS-1 and CTS-4 are less affected by the increase in calcination temperature due to enhanced thermal stability of TiO_2_ with the addition of SiO_2_ (Wei et al., 2014).

### Photocatalytic Activity of TiO_2_-SiO_2_ Composite

**FIGURE 2:**
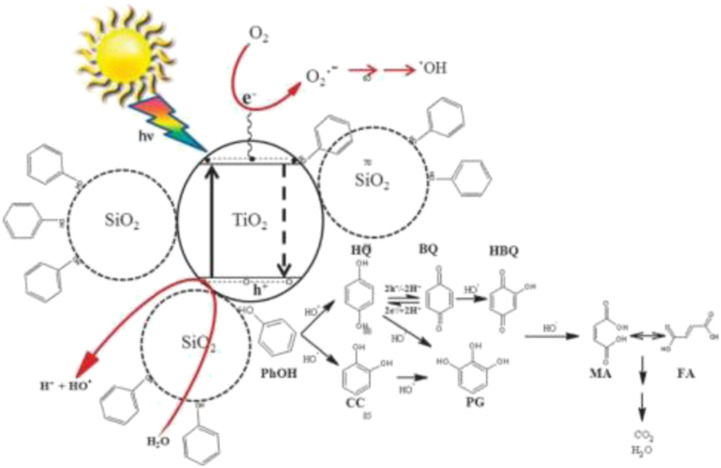
Photocatalytic degradation of phenol over TiO_2_-SiO_2_ photocatalyst (source: Kibombo et al. (2012)).

Binary mixed oxide, TiO_2_-SiO_2_, has been widely documented as a better photocatalyst compared to TiO_2_ alone due to simultaneous roles played by the TiO_2_ and SiO_2_ as photocatalyst and adsorbent, respectively, according to the reaction mechanism shown in Figure 2. The doping of SiO_2_ onto TiO_2_ enhances the adsorption of pollutant molecules to be near the photoactive center of TiO_2_, resulting in more pollutant molecules being broken down, thus producing high degradation rate of pollutant molecules under visible-light region. Due to the presence of more effective adsorption sites in the TiO_2_-SiO_2_ composite system, the photogenerated holes can reach the sites before recombination with electrons thus further enhancing the photocatalytic activity of TiO_2_.

Ramamoorthy et al. (2016) reported that 30% of the TiO_2_ incorporated in TiO_2_-SiO_2_ (200 mg) exhibited 51% degradation of acid orange dye (300 ppm) as compared to only 19% by TiO_2_ (200 mg) after 10 h of visible-light irradiation (150 W tungsten filament lamp). Mungondori et al. (2015) reported an absorption band of 366 nm for TiO_2_ and 397 nm for TiO_2_-SiO_2_ analyzed through shift in absorption band edge. This proves that incorporation of SiO_2_ into TiO_2_ shifts the absorption band of TiO_2_ toward the visible-light region.

When the molar ratio of TiO_2_ in TiO_2_-SiO_2_ was increased, the total surface area of TiO_2_-SiO_2_ decreased causing low photocatalytic activity of the composite catalyst (Ramamoorthy et al., 2016). In a study reported by Jeon et al. (2015), at high molar ratio of TiO_2_ (molar ratio of TiO_2_ precursor to SiO_2_ precursor of 0.05) in the TiO_2_-SiO_2_, dense thin film with mostly blocked pores was observed. This indicated that at higher concentration of TiO_2_ precursor relative to SiO_2_ precursor, the size of the pores became smaller if not completely blocked. In contrast, at high SiO_2_ precursor molar ratio relative to TiO_2_ precursor, the amount of vertically perforated pores increased and formed large micron-sized cracks due to the inability in enduring thermal shrinkage stress as the film thickness increased. Thickness of the film has been reported to decrease with increase in molar ratio of SiO_2_ in TiO_2_/SiO_2_ film (Fateh et al., 2013).

When too small amount of SiO_2_ (<2%) or too large (>5%) is used to modify TiO_2_, it causes lower photocatalytic degradation of TiO_2_-SiO_2_ as compared to commercial P25 TiO_2_. In addition, from the XRD analysis, it was reported that doping a small amount of SiO_2_ on TiO_2_ will not effectively prevent the rutile phase transformation, which also contributes to a lower photocatalytic activity while a high amount of SiO_2_ doping will influence the optical absorption of TiO_2_ which is unfavourable in photocatalytic reactions (Kang et al., 2009). Table 3 presents the gist of the some of the previous studies on TiO_2_-SiO_2_ photocatalyst.

**TABLE 3:** Some of the previous studies on TiO_2_-SiO_2_ photocatalyst.

Photocatalyst	Synthesis method	Target pollutant	Experimental conditions	Results	References
TiO_2_/Cabot-SiO_2,_ TiO_2_/Axim-SiO_2,_ TiO_2_/Fly Ash-SiO_2_	Wet method	2-Propanol	Irradiated with UV 500 W medium pressure Hg lamp with 1.3 mW cm^−2^, 74 µM concentration of 2-propanol	TiO_2_-cabot SiO_2_ completely mineralized 2-propanol (74 µM concentration) in 6 h. TiO_2_/Axim SiO_2_ adsorbed half of 2-propanol (initial conc. 30µM) after 6 h. For TiO_2_/Fly ash SiO_2_ most of the 2-propanol (initial concentration of 30 µM) was adsorbed on the surface	Bellardita et al. (2010)
TiO_2_-SiO_2_	Sol gel	Acid red 88 (*λ*_max_ 505 nm)	Experimental conditions: pH 9, 10–40 ppm acid red 88, 4 h sunlight irradiation, 15 min dark adsorption	TiO_2_: 96.5% removal % TiO_2_-SiO_2_: 94.2% removal %	Balachandran et al. (2014)
TiO_2_-SiO_2_	Sol gel. Rice husk ash as SiO_2_ source	Methyl violet (MV)	Experimental conditions: UV-A and UV-B lamps placed 20 cm above the reactor, H_2_O_2_ as additional oxidant, initial concentration of MV: 1–8ppm	Almost complete degradation of 1 ppm MV in 60 min	Fatimah et al. (2015)
Fibrous nanosilica (KCC-1)-TiO_2_, MCM-41/TiO_2_> SBA-15/TiO_2_	Atomic layer deposition	Methylene blue (1 × 10^–5^ M). Phenol (1 × 10^–5^ M)	75 W of UV light 250–385 nm; 25 mg catalyst, 50 ml reaction solution	MB degradation rate: KCC-1/TiO_2_> MCM-41/TiO_2_> SBA-15/TiO_2._ phenol degradation rate: MCM-41/TiO_2_ > KCC-1/TiO_2_ > SBA-15/TiO_2_	Singh et al. (2016)
SiO_2_-Fe_2_O_3_ (5%)-TiO_2_-B (5%)-N (5%)	Adsorbed-layer nanoreactor synthesis (ANS)	Methyl orange (MO)	4 W and 40 W fluorescent lamp with rare earth phosphor; 5 h of irradiation	Increase in the amount of Fe/Ti from 1% to 8%, decreased the band-gap energy of SiO_2_-TiO_2_-Fe_2_O_3_ from 3.00 to 2.15 eV and decreased the band-gap energy of SiO_2_-Fe_2_O_3_-TiO_2_ from 3.00 to 1.89 eV. Increase in the amount of Fe/Ti increased the photocatalytic degradation of MO under 5 h of 40 W room light irradiation	Wang et al. (2016)

### Challenges in TiO_2_-SiO_2_ in Solar Photocatalysis

Modification of TiO_2_ with SiO_2_ alone is insufficient to maximize the utilization of the TiO_2_ semiconductor in a solar-energy-powered wastewater remediation process. In order to fully utilize the photocatalytic activity of the TiO_2_-SiO_2_ in the visible region, there is a need for third material to be added to the TiO_2_-SiO_2_ in which the third material donates the electrons excited via absorption of photon from sunlight irradiation. Thus, the third material needs to have a low band-gap energy (Eg < 3) in order to utilize the energy directly from the sunlight. Xu et al. (2015) utilized Fe_2_O_3_, which has a band-gap energy of 2.2 eV, to extend the application of TiO_2_ in the visible-light region by coupling with Fe_2_O_3_.

**FIGURE 3:**
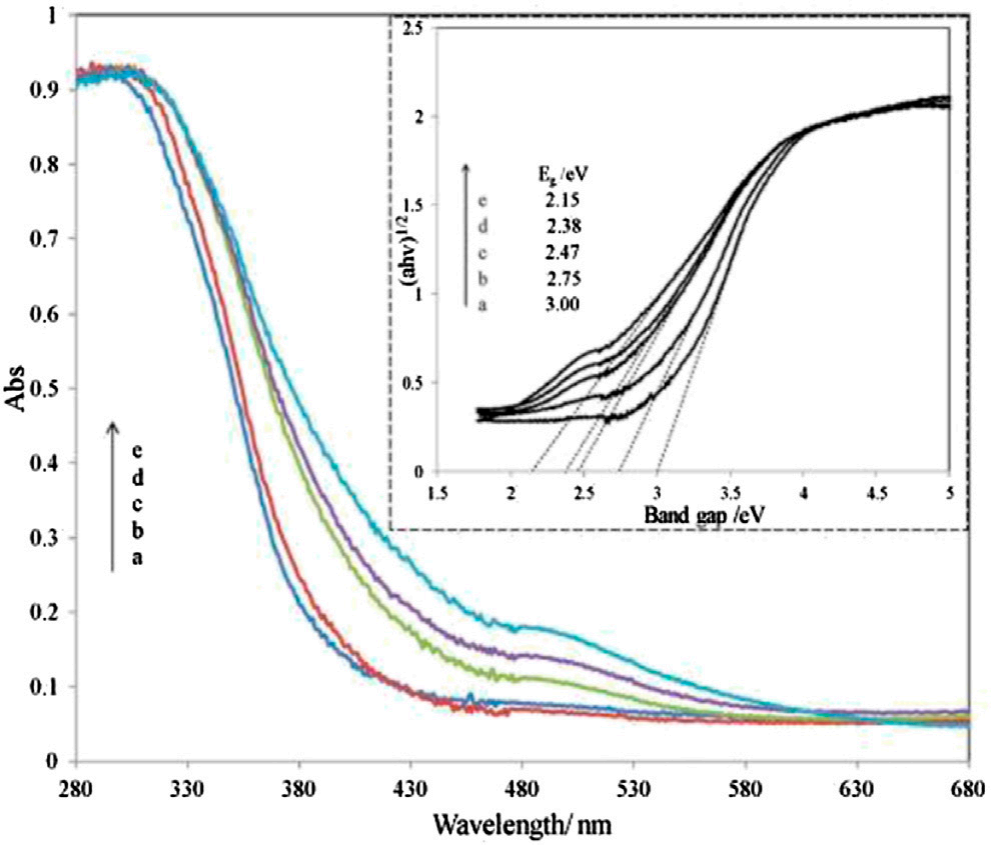
UV-Vis diffuse reflectance spectra of various SiO_2_-TiO_2_-Fe2O3 combinations. Inset: the plots of (αhv)1/2 vs. photon energy of different catalysts. Ratio percentage of Fe/Ti amount: a, 0%; b, 1%; c, 3%; d, 5%; e, 8% (source: Wang et al. (2016)).

**FIGURE 4:**
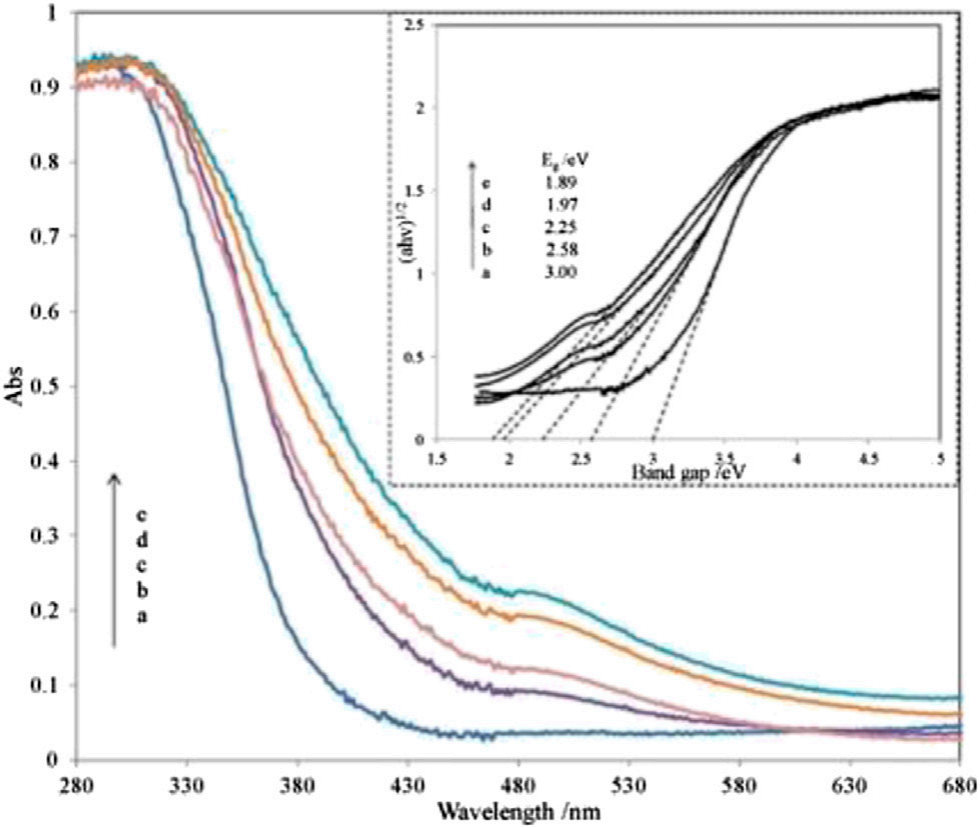
UV-Vis diffuse reflectance spectra of various SiO_2_-Fe_2_O_3_-TiO_2_ combinations. Inset: the plots of (αhv)1/2 vs. the photon energy of different catalysts. Ratio percentage of Fe/Ti amount: a, 0%; b, 1%; c, 3%; d, 5%; e, 8% (source: Wang et al. (2016)).

Wang et al. (2016) reported on utilizing Fe_2_O_3_ coupled TiO_2_-SiO_2_ photocatalyst irradiated under room light to degrade the methyl orange dye. Based on the diffuse reflectance spectra analysis, the absorption edge of SiO_2_-TiO_2_ was approximately 400 nm. However, the absorption edge of SiO_2_-TiO_2_-Fe_2_O_3_ and SiO_2_-Fe_2_O_3_-TiO_2_ is in the visible-light region as shown in Figures 3, 4. This shift directly enables and enhances the photocatalytic activity of TiO_2_-based catalyst in the visible-light region due to the narrowing of the band-gap value. Compared to SiO_2_-TiO_2_-Fe_2_O_2_, SiO_2_-Fe_2_O_3_-TiO_2_ exhibited a higher photocatalytic activity in the visible region due to strong interaction between Fe_2_O_3_ and TiO_2_ and due to the formation of shallow trapping sites for photoinduced electrons and holes.

The codoped SiO_2_-Fe_2_O_3_(5%)-TiO_2_ by N and B exhibited high photocatalytic activity of methyl orange under weak room light irradiation (4 W fluorescent lamp with rare earth phosphor) due to synergistic effect of both N doping and Fe_2_O_3_ coupling which enhanced the visible-light absorbance and reduced the band-gap energy of TiO_2_-based catalyst and at the same time, and both N and B doping on TiO_2_ caused high separation efficiency for photoinduced electrons and holes (Wang et al., 2016).

## Deposition of Plasmonic Metal Nanoparticles

Another option to maximize the application of TiO_2_-SiO_2_ photocatalyst in the visible-light region is by incorporating plasmonic metals such as Ag and Au into TiO_2_ in order to utilize their ability in absorbing strongly in visible region. Their ability in absorbing of solar photons is attributed to the Localized Surface Plasmon Resonance (LSPR) (Hao et al., 2016). LSPR is defined as collective motions of conduction electrons induced by light irradiation (Chen et al., 2012). Some of the previous attempts to deposit plasmonic metal onto the TiO_2_-SiO_2_ composite are listed in Table 4.

**TABLE 4:** Some of the previous studies on plasmonic metal deposited TiO_2_-SiO_2_.

Photocatalyst	Synthesis method	Target pollutant	Result	Experimental conditions	References
AgX-SiO_2_ (X-Cl, Br, or I)		Acetaldehyde (gas phase). Rhodamine B dye (5.4 × 10^–5^ M; liquid phase)	Gas phase: under visible irradiation, photoactivity follows the order AgI-SiO_2_> AgBr-SiO_2_ > AgCl-SiO_2._ under UV irradiation, AgI-SiO_2_ and AgBr-SiO_2_ exhibited similar and higher photocatalytic activity compared to AgCl-SiO_2_. Liquid phase: under visible irradiation, only AgI-SiO_2_ is able to degrade the rhodamine B dye. Under UV irradiation, photocatalytic activity follows the order AgI-SiO_2_> AgBr-SiO_2_ > AgCl-SiO_2_	UV irradiation (320–400 nm). Visible-light irradiation (420 nm); 0.1 g photocatalyst, total volume 150 ml	Hamal and Klabunde (2010)
TiO_2_-SiO_2_-Ag	Biomimetic using lysozyme and sol gel	Rhodamine B (10 ppm)	96.9% of RhB degraded by 0.11%Ag/TiO_2_-SiO_2_, 67.5% by 0.08%Ag/TiO_2_-SiO_2_, and 31% by 0.04% Ag/TiO_2_-SiO_2_ under 4 h visible irradiation (*λ* > 420 nm). Adsorption experiment: 30% RhB adsorbed by TiO_2_-SiO_2_-Ag compared by only 10% for both TiO_2_-Ag and TiO_2_	50 mg of catalyst; 50 ml of 10 ppm RhB, 150 W Xenon arc lamp with cutoff filter (*λ* > 420 nm)	Liu et al. (2013)
Au@SiO_2_-TiO_2_	Sol gel	Methylene blue (2.4 × 10^–5^ M)	After 5 h of irradiation with UV_365nm_ and visible light, TiO_2_ and SiO_2_-TiO_2_ degraded about 44% of MB, Au/TiO_2_ degraded about 80% MB and Au@SiO_2_-TiO_2_ degraded about 95%	UV_365nm._ visible irradiation (400–700 nm) -UV_365nm_, Xenon lamp with filter (400 nm <*λ* < 700 nm); 150 klux	Chen et al. (2012)
SiO_2_/TiO_2_/20%CuBiS_2_/2%Ag	Sol gel Ag deposited via photoinduction. CuBiS_2_ deposited via precipitation	Acid black 1 (10ppm)	Under UV irradiation: 100% degradation of AB1 in 5 min. Under visible-light irradiation: 100% degradation of AB1 in 30 min	Visible irradiation with 150 W incandescent halogen lamp and ultraviolet irradiation with 450 W xenon light with cutoff filter (*λ* > 400 nm)	Abdullah and Kuo (2015)
Ag25/SiO_2_/TiO_2_	Modified Stober process; Au/SiO_2_ and Ag/SiO_2_ deposited onto TiO_2_ by using maleic acid	1.50 × 10^–4^ M aqueous salicylic acid (SA) and 1.50 × 10^–4^ M aniline (A)	Under UV-visible-light irradiation. SA: 3.8 times higher than bare TiO_2._ A: 2.5 times higher than bare TiO_2_	UV-visible: 300 W Xe lamp with water filter to cutoff IR.	Lee et al. (2017a)
SiO_2_-Ag@ TiO_2_	Sol gel; impregnation	10 mg/L of TC 2 × 10^–5^ M of RhB, MB, and MV	60% degradation of TC; 95.9% RhB dye	120 min; visible irradiation, 350 W with UV cut filter (*λ* > 420 nm)	Zhang et al. (2018)
M-TiO_2_/SiO_2_ (M: Pt^4+^, Pd^2+^, and Ag^+^)	Photodeposition method using 8 W blacklight lamp (365 nm) for 15 h in N_2_ atmosphere	Brilliant red K-2G (K-2G) and cationic blue X-GRL (CBX)	300 W high pressure mercury lamp (330–550 nm)	Pt-modified catalyst demonstrated a 2.8 times higher photoactivity than the TiO_2_/SiO_2_ for the photodegradation of K-2G. However, this catalyst had a lower degradation rate for CBX.	Hu et al. (2003)
TiO_2_-SiO_2_ (TS1) materials doped with Ag and Pt nanoparticles	TiO_2_-SiO_2_ was prepared by the sol-gel method. Ag- and Pt-based photocatalysts were prepared by photodeposition (400 W medium pressure mercury lamp)	Phenol	Solar simulator box equipped with a Xe lamp (450 W m^−2^) emitting the solar spectrum	TS1-Ag-1.0 and TS1-Pt-1.0, respectively, showed an increase in the photocatalytic activity up to two and five times higher than TS1	Matos et al. (2018)
Core/shell nanostructures SiO_2_/TiO_2_ doped with Au nanoparticles	A combination of methods to prepared each component	Methyl orange (MO)	The UV light (l < 400 nm) filtered using a UV shield flim (SK-2, Sunnano) to produce only visible light at a power density of 80 ± 2.5 mW cm^−2^	The photocatalyst decomposed 1% of MO solution in 15 ml deionized water in 1 h under the visible light	Lee at al. (2017b)
Ag-coated SiO_2_@TiO_2_ (Ag-SiO_2_@TiO_2_) core-shell nanocomposites	Hydrothermal process and photodeposition (high-pressure Hg UV lamp for 60 min)	Phenol and methylene blue	500 W high-pressure mercury lamp	Ag nanoparticles improved the photocatalytic activity of SiO_2_@TiO_2_ core-shell nanoparticle improved the degradation of phenol and methylene blue	Fu et al. (2019)

**FIGURE 5:**
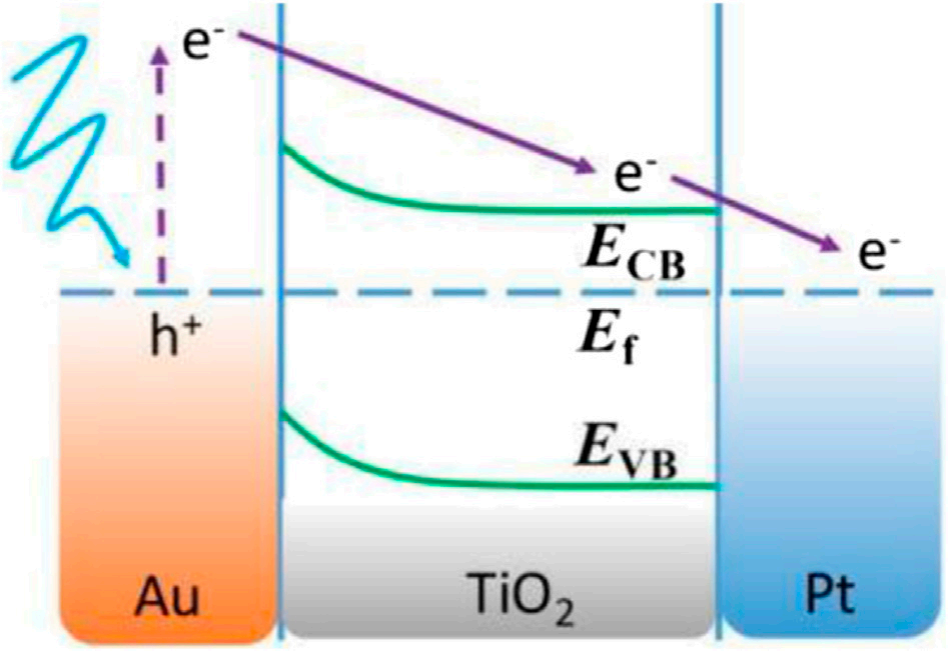
Schematic representations of excited electron generated in AuNS and transfer to the TiO_2_ CB, where ECB, Ef, and EVB represent the energies of the conduction band, Fermi level, and valence band, respectively (source: Shi et al. (2016)).

**FIGURE 6:**
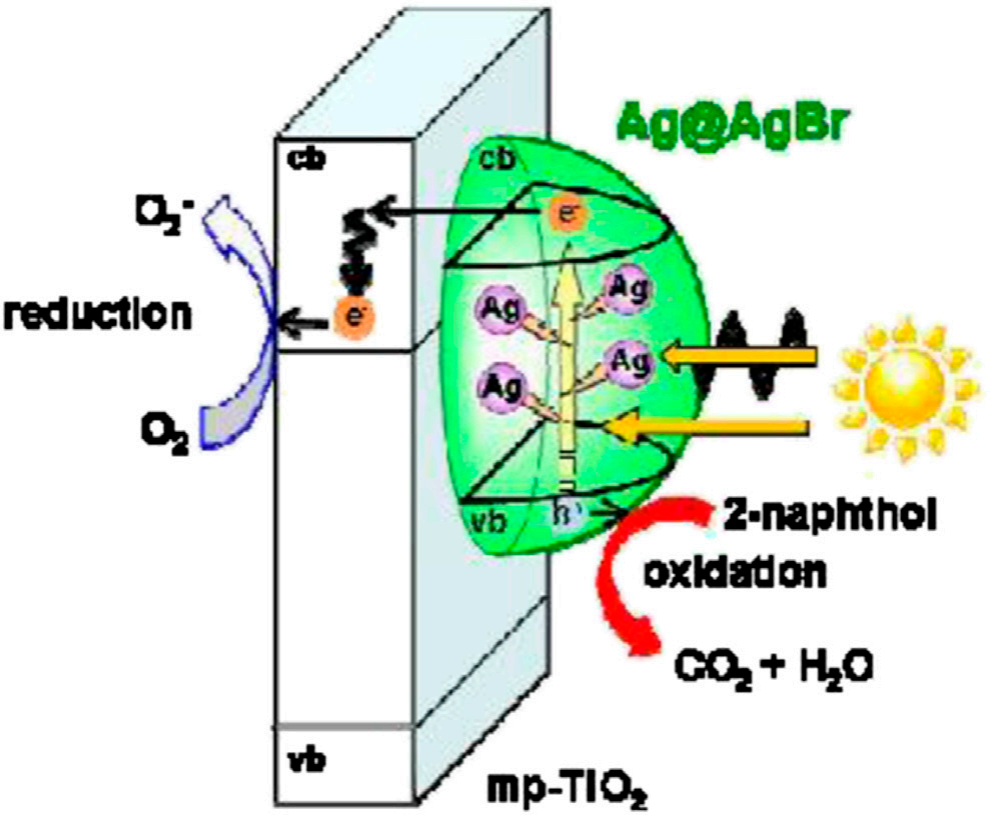
Reaction mechanism of the Ag@AgBr/mp-TiO_2_ photocatalyst under visible-light irradiation (source: Hayashido et al., 2016).

Plasmonic nanoparticles (such as Ag and Au) exhibit a property identified as surface plasmon resonance (SPR) especially in the visible region of the electromagnetic spectrum (Cho and Krishnan, 2013; Abdullah and Kuo, 2015). Many previous research studies deposited Ag/Au nanoparticles onto TiO_2_-SiO_2_ composite photocatalyst due to their ability to enhance photocatalytic activities by trapping the generated photoelectron and reducing the recombination rate of generated electrons and holes by acting as an electron trap (Chen et al., 2012; Abdullah & Kuo, 2015). The Ag/Au nanoparticles enhance the efficiency of TiO_2_-SiO_2_ photocatalyst by absorbing the solar photons and transferring the energetic electron, formed via SPR excitation, into the TiO_2_ (Hamal and Klabunde, 2010; Linic et al., 2011). This mechanism is demonstrated in Figure 5, 6.

Evidence of higher efficiency achieved by plasmonic metal deposited TiO_2_-SiO_2_ was reported by Liu et al. (2013). Liu showed that under 4 h of visible irradiation, the TiO_2_-SiO_2_-Ag photocatalyst exhibited an almost complete degradation of rhodamine B (10ppm) as compared to 80% by TiO_2_-Ag and only 30% by neat TiO_2_. In comparison, Hayashido et al. (2016) documented that Ag@AgBr/mp-TiO_2_ is an efficient photocatalyst in the visible-light region (*λ* > 400 nm) due to the visible-light activity of Ag@AgBr.

**FIGURE 7:**
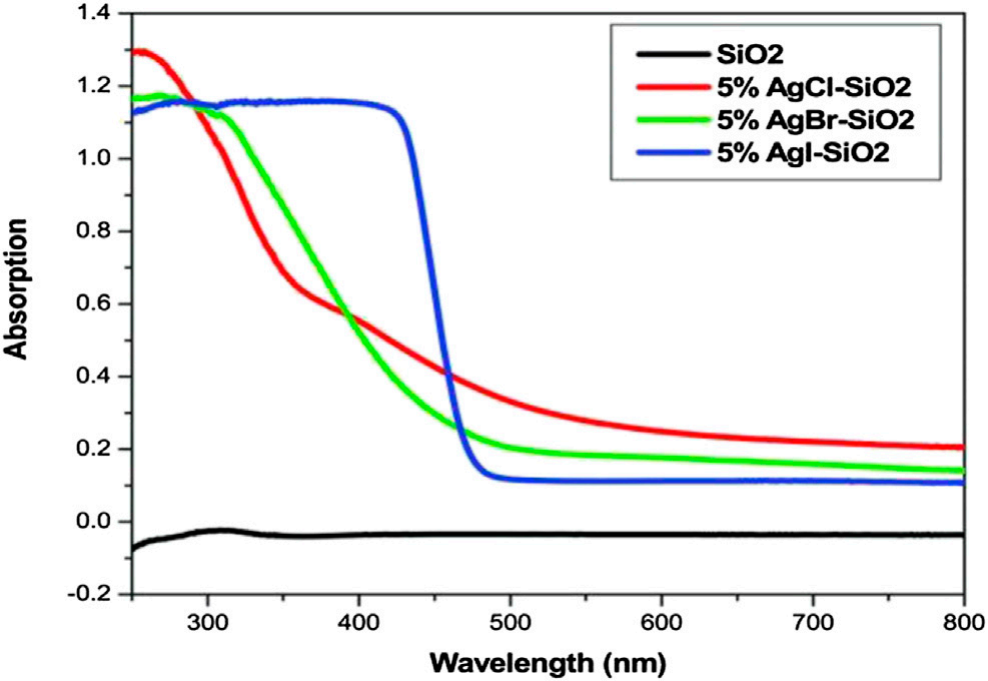
UV-Vis spectrophotometer absorption spectra of 5% silver halide supported on silica (Hamal and Klabunde (2010)).

Addition of Ag into TiO_2_ modifies the lattice parameters of TiO_2_ by generating oxygen vacancies which act as active sites for photocatalysis process, reduces recombination rates of photoexcited electrons and holes due to the formation of Schottky barrier between Ag and TiO_2_, reduces band-gap value, and generates defect site Ti^3+^ (Gupta et al., 2006; Chen et al., 2007; Hamal and Klabunde, 2010). Hamal and Klabunde (2010) documented that 5% AgCl-SiO_2_, 5% AgBr-SiO_2_, and 5% AgI-SiO_2_ exhibited band-gap absorption in visible region as shown in Figure 7 and were able to degrade rhodamine B dye (concentration of 2 × 10^–5^ M) in the liquid phase and acetaldehyde in the gas phase under visible region (*λ* > 420 nm). Among these three, the 5% AgI-SiO_2_ was found to be the best photocatalyst due to its low band gap and high surface area as well as high stability.

When molar ratio of TiO_2_ in the TiO_2_-SiO_2_ photocatalyst structure is increased, the total surface area of TiO_2_-SiO_2_ decreases causing low photocatalytic activity of the composite catalyst (Ramamoorthy et al., 2016). In a study reported by Jeon et al. (2015), at high molar ratio of TiO_2_ (molar ratio of TiO_2_ precursor to SiO_2_ precursor of 0.05) in the TiO_2_-SiO_2_ photocatalyst, a dense thin film with mostly blocked pores was observed. This indicates that at higher concentration of the TiO_2_ precursor relative to the SiO_2_ precursor, the size of the pores becomes smaller and may be completely blocked. In contrast at high SiO_2_ precursor molar ratio relative to TiO_2_ precursor, the amount of vertically perforated pores increases and forms large micron-sized cracks due to the inability in enduring thermal shrinkage stress due to the high film thickness. Thickness of the film has been reported to decrease with the increase in molar ratio of SiO_2_ in TiO_2_/SiO_2_ film (Fateh et al., 2013).

When the amount of SiO_2_ used to modify the TiO_2_ is too small (<2%) or too large (>5%), it causes lower photocatalytic degradation of TiO_2_-SiO_2_ as compared to commercial P25 TiO_2_. In addition, from the XRD analysis, it was reported that doping small amount of SiO_2_ on TiO_2_ will not effectively prevent rutile phase transformation, which also produces lower photocatalytic activity while high mount of SiO_2_ doping will influence the optical absorption of TiO_2_ which is not favourable in photocatalytic reactions (Kang et al., 2009).

Lee et al. (2017a) employed the citrate reduction method to prepare Au and polyol method to prepare Ag nanoparticles, respectively. After that, modified Stober process was employed to coat Au and Ag surfaces with SiO_2_. The prepared composites were later deposited onto TiO_2_ (Degussa P25) by employing maleic acid as an anchoring agent. Lee et al. (2017b) reported that Ag@SiO_2_-doped TiO_2_ nanoparticles are significantly more effective in photocatalytic activity than Ag-doped TiO_2_. Ag25@SiO_2_/TiO_2_ exhibited the highest activity in decomposing aqueous salicylic acid and aniline under UV-visible light irradiation; which was 3.8 and 2.5 times, respectively, that of the bare TiO_2_. The authors attributed the high photocatalytic efficiency of Ag25@SiO_2_/TiO_2_ to strong LSPR effect of Ag.

Zhang et al. (2018) found that SiO_2_-Ag/TiO_2_ (SAT) showed enhanced visible-light activity and UV light activity compared to SiO_2_-TiO_2_/Ag (STA) for degrading tetracycline and traditional dyes. According to them, SiO_2_ serves as an efficient support for the Ag nanoparticle immobilization; meanwhile, TiO_2_ retains the hierarchical structure and prevents agglomeration of Ag nanoparticles during photocatalytic reaction. They attributed the excellent photocatalytic activity of SAT to improve the transport path of photogenerated electrons, diminished recombination probability of electron-hole pairs, and reduced threat of oxidation and corrosion. It should be noted that the SAT retained its photocatalytic efficiency even after five consecutive runs.

## Conclusion

Plasmonic metal particle-incorporated TiO_2_-SiO_2_ composite plays an essential role as a solar photocatalyst which can transform solar energy into chemical energy for application in photocatalysis. Various methods and strategies were presented in this work that highlighted this incorporation, yielding different results. By taking advantage of three synergistic effects that can influence the photocatalytic activity of TiO_2_, ability to absorb solar photons by plasmonic metal nanoparticles (Ag or Au), and high adsorption activity by SiO_2_, it is possible to utilize the renewable solar energy for water and wastewater remediation effectively. With greater focus on this composite photocatalyst, the next few years will bring major advancement in utilizing the Ag/Au-incorporated TiO_2_-SiO_2_ in water and wastewater treatment plants at an industrial scale.
